# The Long-Acting Echinocandin, Rezafungin, Prevents Pneumocystis Pneumonia and Eliminates Pneumocystis from the Lungs in Prophylaxis and Murine Treatment Models

**DOI:** 10.3390/jof7090747

**Published:** 2021-09-11

**Authors:** Melanie T. Cushion, Alan Ashbaugh

**Affiliations:** 1 Department of Internal Medicine, Division of Infectious Diseases, College of Medicine, University of Cincinnati, Cincinnati, OH 45221, USA; alan.ashbaugh@uc.edu; 2 Cincinnati VAMC, Medical Research Service, Cincinnati, OH 45220, USA

**Keywords:** PCP, *Pneumocystis jirovecii* pneumonia (PJP) Pneumocystis, echinocandin, rezafungin, obligate sexual reproduction

## Abstract

Rezafungin is a novel echinocandin in Phase 3 development for prevention of invasive fungal disease caused by *Candida* spp., *Aspergillus* spp. and *Pneumocystis jirovecii* in blood and marrow transplantation patients. For such patients, standard antifungal prophylaxis currently comprises an azole for *Candida* and *Aspergillus* plus trimethoprim-sulfamethoxazole (TMP-SMX) for Pneumocystis pneumonia (PCP) despite drug-drug-interactions and intolerability that may limit their use, thus, alternatives are desirable. Rezafungin demonstrates a favorable safety profile and pharmacokinetic properties that allow for once-weekly dosing in addition, to antifungal activity against these predominant pathogens. Herein, the in vivo effects of rezafungin against *Pneumocystis murina* pneumonia were evaluated in immunosuppressed mouse models of prophylaxis and treatment using microscopy and qPCR assessments. In the prophylaxis model, immunosuppressed mice inoculated with *P. murina* were administered TMP-SMX (50/250 mg/kg 1×/week or 3×/week), caspofungin (5 mg/kg 3×/week), rezafungin (20 mg/kg, 1×/week or 3×/week; 5 mg/kg, 3×/week) intraperitoneally for 2, 4, 6 and 8 weeks, then immunosuppressed for an additional 6 weeks. Rezafungin administered for 4 weeks prevented *P. murina* from developing infection after rezafungin was discontinued. In the treatment model, immunosuppressed mice with *P. murina* pneumonia were treated with rezafungin 20 mg/kg 3×/week intraperitoneally for 2, 4, 6 and 8 weeks. Treatment with rezafungin for 8 weeks resulted in elimination of *P. murina*. Collectively, these studies showed that rezafungin could both prevent infection and eliminate *P. murina* from the lungs of mice. These findings support the obligate role of sexual reproduction for survival and growth of *Pneumocystis* spp. and warrant further investigation for treatment of *P. jirovecii* pneumonia in humans.

## 1. Introduction

*Pneumocystis jirovecii* is an important fungal pathogen in immunocompromised patients [1,2,3,4,5]. Formerly classified as a zoonotic protozoan, *Pneumocystis carinii* is the name of the Pneumocystis species known to infect rats, while *P. jirovecii* is the species infecting humans [6]. *P. jirovecii* can cause severe pneumonia with a high mortality rate especially if untreated [7,8,9]. Owing to the change of nomenclature, the pneumonia can be referred to as PCP or PJP (*Pneumocystis jirovecii* pneumonia) in humans. Patients who are most vulnerable to developing PCP are severely immunocompromised following allogeneic hematopoietic stem cell transplantation or solid organ transplantation, those with hematological malignancies undergoing immunosuppressive chemotherapy, as well as patients with rheumatologic disorders and those with HIV infection (CD4 count ˂ 200 cells/µL) [2,3,4,10]. Among patients hospitalized for PCP, the most prevalent host factors were malignancies (46%; 61% of the malignancies were hematologic) and HIV infection (18%) [11].

The current recommendation for first-line prophylaxis against PCP is trimethoprim-sulfamethoxazole (TMP-SMX) and is used in high-risk patients throughout the time of treatment-induced immunosuppression or until the CD4 count increases to above 200 cells/µL [2,3,5,12,13]. Prophylaxis is recommended to continue from engraftment or post-transplant for ≥6 months [3,12]. Prolonged prophylaxis for PCP following solid organ transplantation has been suggested in patients at a higher risk of developing late cases of PCP [1,14,15,16].

Although the efficacy of TMP-SMX is well established, there are concerns for its safety and tolerability, such as bone marrow suppression, liver toxicity and severe skin reactions [12,13]. In an open-label randomized controlled study, up to 42% of patients receiving TMP-SMX for PCP prophylaxis discontinued therapy due to adverse events [17]. Options for second-line prophylaxis, such as aerosolized pentamidine, dapsone and atovaquone, should be considered only if a patient is intolerant to or experiences a severe adverse event from TMP-SMX. None of these agents have been compared with TMP-SMX as first-line prophylaxis in a randomized controlled trial and considerations with their use include cost, bone marrow toxicity, route and frequency of administration and the need for additional prophylactic coverage [12].

Rezafungin is a novel echinocandin currently, in Phase 3 development for prophylaxis of invasive fungal disease in blood and marrow transplantation (ReSPECT NCT04368559). While rezafungin antifungal activity covers a broad-spectrum of *Candida* and *Aspergillus* spp. similar to that of currently approved echinocandins [18,19,20], it is notable for also demonstrating excellent activity against Pneumocystis species (spp.) comparable to that of TMP-SMX, as observed in biofilms and animal models [21,22,23]. Rezafungin demonstrates a long half-life (~130 h in humans) and safety profile that, as previously reported, refs. [24,25] support front-loaded, once-weekly dosing. Collectively, these data suggest the potential for a simplified, single-agent antifungal prophylaxis regimen with rezafungin.

The echinocandins are a newer class of antifungal agents and currently approved echinocandins have had mixed responses in patients with PCP [26,27]. The mechanism of action, inhibition of β-1,3-D-glucan (BG) synthesis, could explain the disparate efficacy in patients, as *Pneumocystis* spp. have two distinct life cycle stages: the trophic forms, which do not express BG and the asci, which contain this polymerized sugar in their cell wall [28]. *Pneumocystis* spp. have been thought to replicate asexually via binary fission of the trophic forms and sexually through a mating process resulting in asci [29]. Recent reports have questioned whether the asexual mode is always operational and suggest these fungi may rely solely on sexual reproduction to proliferate [30]. In studies of murine PCP, it was shown that asci disappear from the infected mouse lungs after 3 weeks of treatment, while large numbers of non-BG expressing organisms remain, illustrating the differential effect on these life cycle stages [28].

In previous studies with rezafungin in immunosuppressed mouse models of prophylaxis and treatment, we reported the inhibition of growth (but not clearance) of all life cycle stages of *P. murina* [22,23]. Thus, the objectives of the present study were 2-fold: to identify the precise dose and duration of rezafungin (1) that completely prevented infection in the prophylactic mouse model and (2) that eliminated established infection in the treatment mouse model of PCP.

## 2. Materials and Methods

These studies were performed in accordance with the Guide for the Care and Use of Laboratory Animals, 8th ed. (National Academies Press, Washington, DC, USA), in AAALAC-accredited laboratories under the supervision of veterinarians. In addition, all procedures were conducted in compliance with the Institutional Animal Care and Use Committee at the Veterans Affairs Medical Center, Cincinnati, OH, USA.

To safeguard against environmental exposure of *P. murina* and other microbes, mice were housed under barrier conditions with autoclaved food; acidified water (sulfuric acid 1 mL/L) and bedding sterilized in shoebox cages equipped with sterile microfilter lids. Pathogenic bacteria cannot grow in acidified water (pH between 2.5 and 3.0) and many research institutes and universities, as well as The Jackson Laboratory, use acidified water for their mice [31]. Access was limited to animal care and technical personnel who are required to wear sterile caps, gowns, gloves, and booties while in the animal rooms. The animals are observed daily and those that appear gravely ill or moribund are routinely euthanized by a method approved by the AVMA Panel on Euthanasia.

Test agents were supplied by Cidara Therapeutics, Inc. (San Diego, CA, USA). Sulfamethoxazole/Trimethoprim (TMP-SMX) was supplied by Aurobindo Pharma USA (Dayton, NJ, USA). Caspofungin (CAS) was supplied by Merck & Co (Whitehouse Plains, NJ, USA).

### 2.1. PCP Prophylaxis Mouse Model

**Scheme 1:**
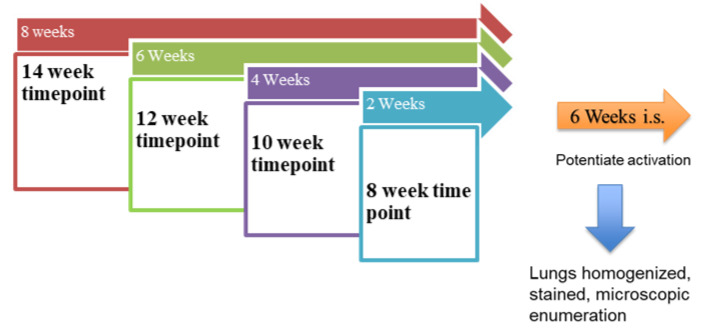
Prophylaxis Model. The arrows above the boxes represent the length of drug administration. After each treatment period, the mice were kept on immunosuppression for 6 weeks. The boxes indicate the timepoints. “i.s” = immunosuppression.

(Refer to Scheme 1, below). C3H/HeN mice (Charles River, Wilmington, MA, USA) weighing 20 g were immunosuppressed by adding dexamethasone (4 mg/L) to acidified drinking water. The mice were infected with *P. murina* organisms isolated from previously infected mice and stored in liquid nitrogen (Cincinnati VAMC, Cincinnati, OH, USA). Such isolates are tested for microbial contamination and ATP content before use as quality control methods [32]. A dose of 2 × 10^6^/50 µL was instilled by intranasal inoculation along with the concurrent initiation of prophylactic drug treatments. Inoculation of a set number of organisms provides a consistent infection among the groups of mice. Given the slow growing nature of *P. murina*, drug administration at the time of inoculation models prophylactic administration. Seven groups of mice received one of the following regimens administered intraperitoneally (IP): corticosteroid as negative control, TMP-SMX (50/250 mg/kg, 1×/week or 3×/week) as positive control dosed at the same times as the other test agents, CAS (5 mg/kg, 3×/week) as comparator echinocandin, or rezafungin (20 mg/kg, 1×/week or 3×/week; 5 mg/kg, 3×/week). The control group included 10 mice and each prophylaxis regimen included 40 mice. Prophylaxis was continued for 2, 4, 6 and 8 weeks (n = 10 for each timepoint). A total of 10 mice per group were needed to detect statistically significant differences between groups. Following discontinuation of treatment, mice were immunosuppressed for an additional 6 weeks, allowing for reactivation of any residual *P. murina*. Mice were then euthanized by CO_2_ and the lungs were processed for analysis by homogenization. Dilutions of the lung homogenates were dropped on pre-etched slides (10 µL × 3 drops per slide) stained with Diff-Quik and Cresyl Echt Violet (CEV) and microscopically enumerated (30 Oil Immersion Fields at 1250×) to quantify the nuclei and asci forms of the fungus, respectively [28].

Of note, CAS 5 mg/kg and rezafungin 20 mg/kg doses were selected for this study because they are considered the human equivalent doses in mice. The AUC/C_max_ of CAS 5 mg/kg in mice exceeds that in human [33,34] and serves as a good comparator to rezafungin if administered using the same 3×/week dosing regimen. The 3×/week frequency of rezafungin was chosen because the half-life is shorter in mice (~25 h) [35] versus humans (~130 h) and this dosing regimen would approximate the AUC achieved in humans while compensating for the shorter half-life in mice.

### 2.2. PCP Treatment Mouse Model 

**Scheme 2:**
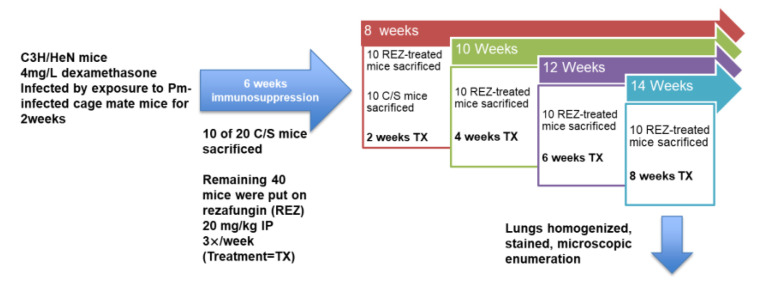
*PCP Treatment Model*. After exposure to *P. murina* (Pm) and immunosuppression for 6 weeks, 10 control (untreated, “C/S”) mice were sacrificed to evaluate organism burden. Forty mice were randomized and rezafungin treatment was initiated. After 2 weeks of treatment (red box and “8 weeks” arrow) 10 mice were sacrificed as well as the remaining C/S mice. Lungs were homogenized and evaluated for organism burden. Time points are indicated by the arrows on top of the boxes. Treatment length (TX) is indicated in each box (2-, 4-, 6-, 8 weeks).

(Refer to Scheme 2, below) C3H/HeN mice (Charles River) (20 g) were infected with *P. murina* through exposure to seed mice (those with fulminant infection) by rotation into the cages for 2 weeks. Mice were immunosuppressed by adding dexamethasone (4 mg/L) to acidified drinking water. The exposure method is a more natural route of infection and can be used in treatment studies because sentinel mice are checked for the presence of standard moderate infections prior to treatment.

Sixty mice received the immunosuppressant and after 6 weeks of immunosuppression, 10 of the 20 mice that were to receive no treatment (controls) were sacrificed and evaluated for infection. At the same time, the remaining 40 mice were put on rezafungin at 20 mg/kg IP 3×/week for up to 8 weeks of treatment. After 2 weeks of rezafungin treatment (week 8 time point; 6 weeks of immunosuppression + 2 weeks of treatment), 10 of the immunosuppressed, rezafungin-treated mice and the remaining untreated controls (10 mice) were sacrificed. Infected and untreated mice do not survive past this time point. After 4 weeks of rezafungin treatment (week 10 time point), 10 mice were sacrificed and evaluated for infection and after 6 weeks of treatment (week 12 time point), another 10 mice were evaluated. At the final week 14 time point, the remaining 10 mice had received 8 weeks of the rezafungin regimen and were sacrificed. Mice were euthanized by CO_2_ inhalation and the lungs were processed for analysis by homogenization. Lung homogenates were microscopically analyzed for organism burdens as described above.

Further analysis by RT-qPCR used total RNA isolated from the lung homogenates with TRIZOL reagent and transcribed to cDNA to quantify *P. murina* ribosomal RNA gene message levels. RT-qPCR was performed using a previously published TaqMan assay [36].

The threshold cycle for each sample was defined as the point at which fluorescence generated by degradation of the TaqMan probe showed a significant increase above baseline. A standard curve using a dilution series of cDNA made from RNA isolated from 10^7^ *P. murina* nuclei was generated to convert the threshold cycle data to *P. murina* nuclei. This standard curve was used to estimate the level of infection. 

### 2.3. Statistical Analysis 

The following statistical analyses were performed for both models (PCP prophylaxis and treatment models). The nuclei and asci count for each lung were log transformed and analyzed by analysis of covariance to determine statistical significance (*p* ˂ 0.05). Individual groups were compared with the Student–Newman–Keuls *t* test for multiple comparisons. Survival curves were based on the 98-day treatment period (GraphPad Prism v9).

## 3. Results

### 3.1. PCP Prophylaxis Mouse Model

**Figure 1:**
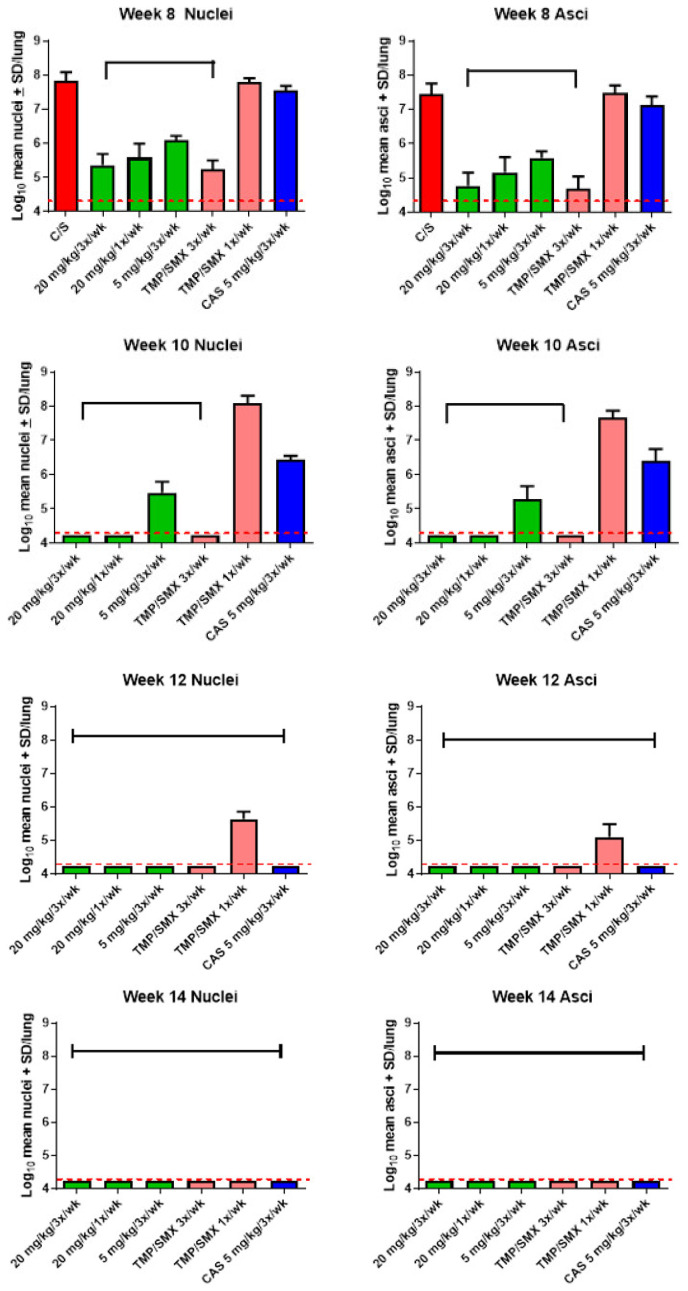
Log10 mean nuclei and asci counts after 2 to 8 weeks of prophylactic treatment followed by additional 6 weeks of immunosuppression without treatment. Color code: red = C/S; vehicle treated negative control; green = rezafungin; pink = T/S (trimethoprim/sulfamethoxazole), blue = CAS (caspofungin). Bracket denotes significant difference from week 8 C/S group and CAS group (*p* < 0.05). Capped line indicates significant difference from week 8 C/S group. Red dotted line indicates limit of microscopic detection (log_10_ 4.24).

**Figure 2:**
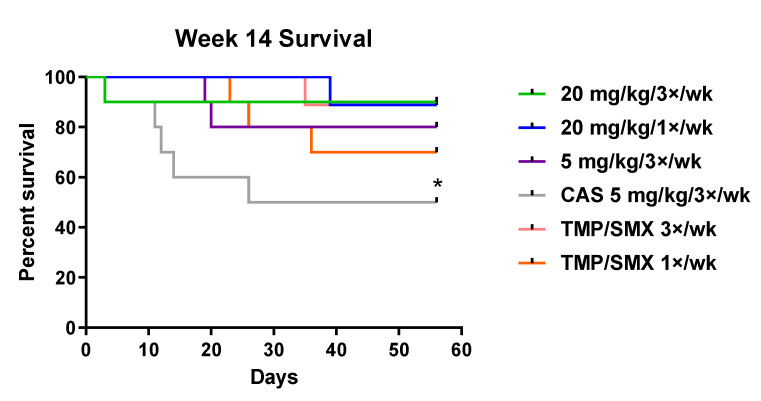
Survival curves for rezafungin and comparators at Week 14 after 56 treatment days of study. CAS = caspofungin; TMP/SMX = trimethoprim/sulfamethoxazole. * Significant difference between rezafungin 20 mg/kg/3×/week (green line) and CAS (grey line) at week 14 (*p* < 0.05).

Significant reductions in both nuclei and asci burdens at all timepoints were observed in all mice that received rezafungin as prophylaxis compared with negative control (vehicle) mice at week 8 (Figure 1). Following 4 weeks of rezafungin prophylaxis (plus 6 weeks of additional immunosuppression; week 10 timepoint), rezafungin 20 mg/kg (1×/week or 3×/week) prevented *P. murina* from activating an infection. Further, 6 and 8 weeks of rezafungin prophylaxis (week 12 and 14 timepoints) prevented reactivation of *P. murina* in 100% of the mice. Significant differences in nuclei and asci counts between each group given rezafungin and caspofungin (CAS; comparator echinocandin control) were observed at 2 and 4 weeks of treatment (week 8 and 10 timepoints, respectively). Rezafungin prophylaxis resulted in significantly lower nuclei and asci counts at 2, 4 and 6 weeks (week 8, 10 and 12 timepoints) compared with TMP-SMX 1×/week (positive treatment control). At week 14, improved survival was observed in the rezafungin 20 mg/kg 3×/week prophylaxis group compared with CAS after 56 days of treatment (Figure 2).

### 3.2. PCP Treatment Mouse Model

**Figure 3:**
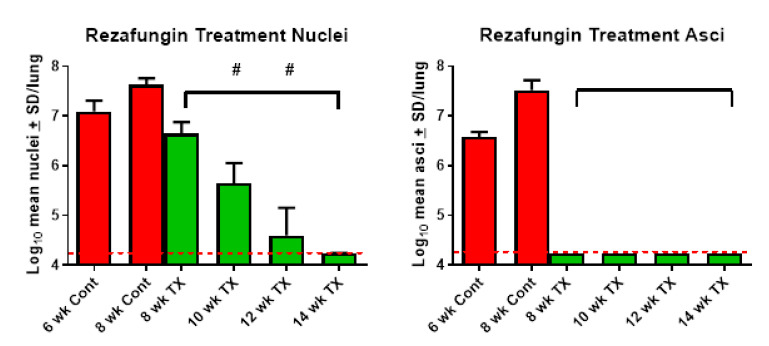
Treatment with rezafungin eliminates *P. murina*. Log_10_ mean nuclei and asci counts after 2 to 8 weeks of rezafungin treatment. Color code: red = vehicle treated control bars; green = rezafungin-treated 20 mg/kg/3×/week. Bracket denotes significant difference from 8-week control (*p* < 0.05). # denotes significant difference from previous week timepoint. Red dotted line indicates limit of detection (log_10_ 4.24).

Significant reductions in nuclei and asci counts were observed after 2 weeks of rezafungin treatment (8-week study duration) compared with the control group (Figure 3). No asci were present at any of the timepoints in the rezafungin-treated mice and significant reductions in nuclei were observed at all timepoints compared with the control group. Significant reductions in nuclei were observed from week 8 to week 10 and from week 10 to week 12, with no nuclei present at week 14. There was no significant difference in survival between the control group and rezafungin groups. 

**Figure 4:**
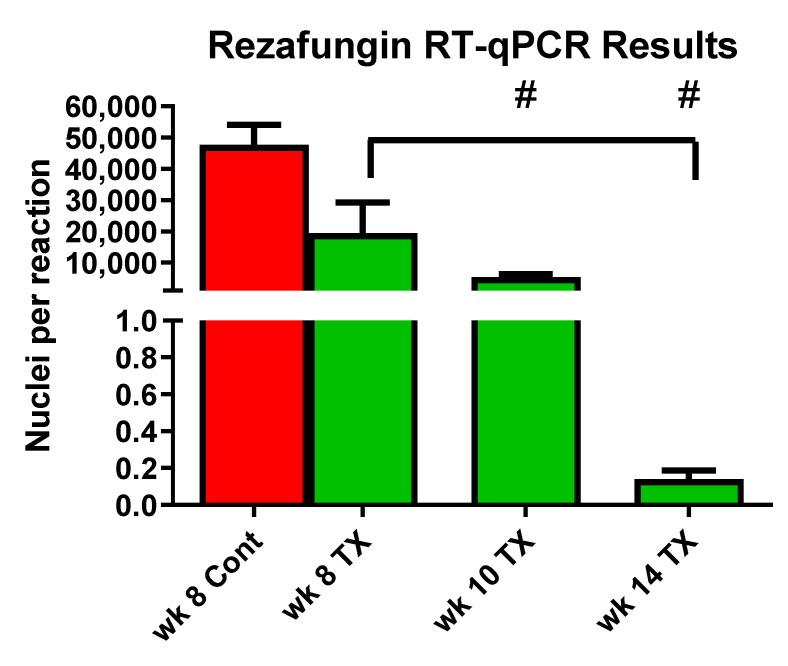
RT-qPCR data from cDNA isolated from lung homogenates and quantified using a TaqMan Assay. Color code: red = vehicle treated control bars; green = rezafungin-treated 20 mg/kg/3×/week. TX = Treatment. Bracket denotes significant difference from 8-week control. # denotes significant difference from previous week timepoint. A 14 week signal is below detection.

Analysis by quantitative reverse transcription PCR (RT-qPCR) showed significant reductions of signals at all timepoints in the rezafungin groups compared with the week 8 control group (Figure 4). At the 14-week time point, no signal was detected, indicating elimination of the infection.

## 4. Discussion

Echinocandins inhibit the synthesis of BG, an essential component of the fungal cell wall [23] found in the asci form of *Pneumocystis* spp. In previous studies using the mouse model of PCP, anidulafungin was shown to halt the sexual cycle of *P. murina* and *P. carinii* by inhibiting the synthesis of BG in the ascus form; however, significant numbers of non-asci forms remained in the lungs [28,37]. Notably, mice without asci due to anidulafungin treatment, were unable to transmit the infection. These studies provided evidence that the ascus is the agent of transmission for *Pneumocystis* spp. and is needed for a productive infection. In addition, echinocandin discontinuation along with continued immunosuppression allowed the asci to repopulate the lungs, showing that those organisms remaining after anidulafungin treatment were viable and able to reactivate the infection [28].

In contrast, an early in vivo study evaluating rezafungin as prophylaxis with TMP-SMX as the active control in a mouse model of PCP showed that rezafungin statistically significantly reduced nuclei and asci counts of *P. murina* at all doses tested (20 mg/kg, 1×/week or 3×/week and 5 mg/gkg 3×/week) and demonstrated in vivo efficacy (20). Furthermore, in the present studies, prophylaxis with rezafungin for as short as 4 weeks prevented *P. murina* organisms from developing an infection after rezafungin had been discontinued and animals were immunosuppressed for an additional 6 weeks, more effectively than CAS.

Data from the treatment mouse model study showed that rezafungin inhibits asci and consequently sexual reproduction in a manner dependent on treatment duration. It can also be surmised that *P. murina* cannot be sustained by alternative means of replication, such as asexual reproduction, in the absence of asci during an extended treatment period with rezafungin.

With the increasing complexity of azole-based prophylaxis (e.g., drug-drug interactions), echinocandins have garnered attention for their potential in antifungal prophylaxis [38,39]. However, despite limited alternatives to standard TMP-SMX, micafungin, anidulafungin and CAS have not been recommended for prophylaxis against PCP based on in vivo efficacy [26,40,41,42]. The PK profile of rezafungin not only sets it apart from other echinocandins in traditional PK analysis; higher penetration of rezafungin at the infected tissue site, as observed compared with micafungin, may explain observed differences in efficacy compared with other echinocandins [23].

Rezafungin is the first antifungal agent to be studied as a single agent for prophylaxis against *Candida* spp., *Aspergillus* spp. and *P. jirovecii*. Rezafungin has demonstrated in vivo efficacy comparable to that of TMP-SMX and, furthermore, lacks the safety concerns associated with TMP-SMX. While our study was limited to nonclinical models, clinical evaluation of rezafungin to date has demonstrated its safety and efficacy. Rezafungin was safe and well tolerated in single- and multiple-dose studies of intravenous rezafungin administered up to 400 mg once weekly [24,43,44], as well as in the completed Phase 2 trial of rezafungin for treatment of candidemia and invasive candidiasis (STRIVE; NCT02734862) [44]. In the ongoing phase 3, prospective, randomized, double-blind trial of rezafungin for prevention of invasive fungal disease caused by *Candida*, *Aspergillus* and *P. jirovecii* in patients undergoing allogeneic blood and marrow transplantation (ReSPECT; NCT04368559), the efficacy and safety of once-weekly IV rezafungin will be evaluated compared with standard of care (once-daily azole plus TMP-SMX).

The in vivo preclinical data reported herein demonstrate the excellent activity of rezafungin against *P. murina* and support its ongoing development as a potential new approach to antifungal prophylaxis and therapy.

## Data Availability

All data are reported in this study.

## References

[1] Brakemeier S., Pfau A., Zukunft B., Budde K., Nickel P. (2018). Prophylaxis and treatment of *Pneumocystis jirovecii* pneumonia after solid organ transplantation. Pharmacol. Res..

[2] Cordonnier C., Cesaro S., Maschmeyer G., Einsele H., Donnelly J.P., Alanio A., Hauser P.M., Lagrou K., Melchers W.J., Helweg-Larsen J. (2016). *Pneumocystis jirovecii* pneumonia: Still a concern in patients with haematological malignancies and stem cell transplant recipients. J. Antimicrob. Chemother..

[3] Fishman J.A., Gans H. (2019). *Pneumocystis jiroveci* in solid organ transplantation: Guidelines from the American Society of Transplantation Infectious Diseases Community of Practice. Clin. Transplant..

[4] Salzer H.J.F., Schafer G., Hoenigl M., Gunther G., Hoffmann C., Kalsdorf B., Alanio A., Lange C. (2018). Clinical, Diagnostic, and Treatment Disparities between HIV-Infected and Non-HIV-Infected Immunocompromised Patients with *Pneumocystis jirovecii* Pneumonia. Respiration.

[5] Sokulska M., Kicia M., Wesolowska M., Hendrich A.B. (2015). *Pneumocystis jirovecii*—From a commensal to pathogen: Clinical and diagnostic review. Parasitol. Res..

[6] Redhead S.A., Cushion M.T., Frenkel J.K., Stringer J.R. (2006). Pneumocystis and *Trypanosoma cruzi: Nomenclature* and typifications. J. Eukaryot. Microbiol..

[7] Truong J., Ashurst J.V. (2021). Pneumocystis jirovecii Pneumonia.

[8] Delliere S., Gits-Muselli M., Bretagne S., Alanio A. (2020). Outbreak-Causing Fungi: *Pneumocystis jirovecii*. Mycopathologia.

[9] Cilloniz C., Dominedo C., Alvarez-Martinez M.J., Moreno A., Garcia F., Torres A., Miro J.M. (2019). Pneumocystis pneumonia in the twenty-first century: HIV-infected versus HIV-uninfected patients. Expert Rev. Anti. Infect. Ther..

[10] Tabanor J.A., Lakshminarayanan S. (2019). Do patients on biologic drugs for rheumatic disease need PCP prophylaxis?. Cleve. Clin. J. Med..

[11] Kanj A., Samhouri B., Abdallah N., Chehab O., Baqir M. (2021). Host Factors and Outcomes in Hospitalizations for *Pneumocystis jirovecii* Pneumonia in the United States. Mayo Clin. Proc..

[12] Maertens J., Cesaro S., Maschmeyer G., Einsele H., Donnelly J.P., Alanio A., Hauser P.M., Lagrou K., Melchers W.J., Helweg-Larsen J. (2016). ECIL guidelines for preventing *Pneumocystis jirovecii* pneumonia in patients with haematological malignancies and stem cell transplant recipients. J. Antimicrob. Chemother..

[13] Redjoul R., Robin C., Foulet F., Leclerc M., Beckerich F., Cabanne L., di Blasi R., Pautas C., Toma A., Botterel F. (2019). *Pneumocystis jirovecii* pneumonia prophylaxis in allogeneic hematopoietic cell transplant recipients: Can we always follow the guidelines?. Bone Marrow Transpl..

[14] Choi Y.I., Hwang S., Park G.C., Namgoong J.M., Jung D.H., Song G.W., Ha T.Y., Moon D.B., Kim K.H., Ahn C.S. (2013). Clinical outcomes of Pneumocystis carinii pneumonia in adult liver transplant recipients. Transplant. Proc..

[15] Iriart X., Belval T.C., Fillaux J., Esposito L., Lavergne R.A., Cardeau-Desangles I., Roques O., del Bello A., Cointault O., Lavayssiere L. (2015). Risk factors of Pneumocystis pneumonia in solid organ recipients in the era of the common use of posttransplantation prophylaxis. Am. J. Transpl..

[16] Wang E.H., Partovi N., Levy R.D., Shapiro R.J., Yoshida E.M., Greanya E.D. (2012). Pneumocystis pneumonia in solid organ transplant recipients: Not yet an infection of the past. Transpl. Infect. Dis..

[17] Utsunomiya M., Dobashi H., Odani T., Saito K., Yokogawa N., Nagasaka K., Takenaka K., Soejima M., Sugihara T., Hagiyama H. (2020). An open-label, randomized controlled trial of sulfamethoxazole-trimethoprim for Pneumocystis prophylaxis: Results of 52-week follow-up. Rheumatol. Adv. Pract..

[18] Wiederhold N.P., Locke J.B., Daruwala P., Bartizal K. (2018). Rezafungin (CD101) demonstrates potent in vitro activity against Aspergillus, including azole-resistant *Aspergillus fumigatus* isolates and cryptic species. J. Antimicrob. Chemother..

[19] Pfaller M.A., Carvalhaes C., Messer S.A., Rhomberg P.R., Castanheira M. (2020). Activity of a Long-Acting Echinocandin, Rezafungin, and Comparator Antifungal Agents Tested against Contemporary Invasive Fungal Isolates (SENTRY Program, 2016 to 2018). Antimicrob. Agents Chemother..

[20] Toth Z., Forgacs L., Locke J.B., Kardos G., Nagy F., Kovacs R., Szekely A., Borman A.M., Majoros L. (2019). In vitro activity of rezafungin against common and rare Candida species and *Saccharomyces cerevisiae*. J. Antimicrob. Chemother..

[21] Cushion M.T., Collins M.S., Locke J.B., Ong V., Bartizal K. (2018). Novel Once-Weekly Echinocandin Rezafungin (CD101) Prevention and Treatment of Pneumocystis Biofilms.

[22] Miesel L., Cushion M.T., Ashbaugh A., Lopez S.R., Ong V. (2021). Efficacy of Rezafungin in Prophylactic Mouse Models of Invasive Candidiasis, Aspergillosis, and Pneumocystis Pneumonia. Antimicrob. Agents Chemother..

[23] Zhao Y., Perlin D.S. (2020). Review of the Novel Echinocandin Antifungal Rezafungin: Animal Studies and Clinical Data. J. Fungi.

[24] Ham Y.Y., Lewis J.S., Thompson G.R. (2021). Rezafungin: A novel antifungal for the treatment of invasive candidiasis. Future Microbiol..

[25] Sofjan A.K., Mitchell A., Shah D.N., Nguyen T., Sim M., Trojcak A., Beyda N.D., Garey K.W. (2018). Rezafungin (CD101), a next-generation echinocandin: A systematic literature review and assessment of possible place in therapy. J. Glob. Antimicrob. Resist..

[26] Huang Y.S., Liu C.E., Lin S.P., Lee C.H., Yang C.J., Lin C.Y., Tang H.J., Lee Y.C., Lin Y.C., Lee Y.T. (2019). Taiwan, Echinocandins as alternative treatment for HIV-infected patients with Pneumocystis pneumonia. AIDS.

[27] Huang Y.-S., Yang J.-J., Lee N.-Y., Chen G.-J., Ko W.-C., Sun H.-Y., Hung C.-C. (2017). Treatment of *Pneumocystis jirovecii* pneumonia in HIV-infected patients: A review. Expert Rev. Anti-Infect. Ther..

[28] Cushion M.T., Linke M.J., Ashbaugh A., Sesterhenn T., Collins M.S., Lynch K., Brubaker R., Walzer P.D. (2010). Echinocandin treatment of pneumocystis pneumonia in rodent models depletes cysts leaving trophic burdens that cannot transmit the infection. PLoS ONE.

[29] Cushion M.T. (2010). Stealth and opportunism: Alternative lifestyles of species in the fungal genus Pneumocystis. Mol. Genet. Genom. MGG.

[30] Hauser P.M., Cushion M.T. (2018). Is sex necessary for the proliferation and transmission of Pneumocystis?. PLoS Pathog..

[31] Whipple B., Agar J., Zhao J., Pearce D.A., Kovács A.D. (2021). The acidified drinking water-induced changes in the behavior and gut microbiota of wild-type mice depend on the acidification mode. Sci. Rep..

[32] Collins M.S., Cushion M.T. (2001). Standardization of an in vitro drug screening assay by use of cryopreserved and characterized *Pneumocystis carinii* populations. J. Eukaryot. Microbiol. Suppl..

[33] Andes D., Diekema D.J., Pfaller M.A., Bohrmuller J., Marchillo K., Lepak A. (2010). In vivo comparison of the pharmacodynamic targets for echinocandin drugs against Candida species. Antimicrob. Agents Chemother..

[34] Merck & Co., Inc. (2001). Cancidas Prescribing Information.

[35] Lepak A.J., Zhao M., VanScoy B., Ambrose P.G., Andes D.R. (2018). Pharmacodynamics of a Long-Acting Echinocandin, CD101, in a Neutropenic Invasive-Candidiasis Murine Model Using an Extended-Interval Dosing Design. Antimicrob. Agents Chemother..

[36] Ruan S., Tate C., Lee J.J., Ritter T., Kolls J.K., Shellito J.E. (2002). Local delivery of the viral interleukin-10 gene suppresses tissue inflammation in murine *Pneumocystis carinii* infection. Infect. Immun..

[37] Cushion M.T., Ashbaugh A., Hendrix K., Linke M.J., Tisdale N., Sayson S.G., Porollo A. (2018). Gene Expression of *Pneumocystis murina* after Treatment with Anidulafungin Results in Strong Signals for Sexual Reproduction, Cell Wall Integrity, and Cell Cycle Arrest, Indicating a Requirement for Ascus Formation for Proliferation. Antimicrob. Agents Chemother..

[38] Epstein D.J., Seo S.K., Brown J.M., Papanicolaou G.A. (2018). Echinocandin prophylaxis in patients undergoing haematopoietic cell transplantation and other treatments for haematological malignancies. J. Antimicrob. Chemother..

[39] Brüggemann R.J.M., Alffenaar J.-W.C., Blijlevens N.M.A., Billaud E.M., Kosterink J.G.W., Verweij P.E., Burger D.M., Saravolatz L.D. (2009). Clinical Relevance of the Pharmacokinetic Interactions of Azole Antifungal Drugs with Other Coadministered Agents. Clin. Infect. Dis..

[40] Wang M., Lang G., Chen Y., Hu C., Guo Y., Tao R., Dong X., Zhu B. (2019). A Pilot Study of Echinocandin Combination with Trimethoprim/Sulfamethoxazole and Clindamycin for the Treatment of AIDS Patients with Pneumocystis Pneumonia. J. Immunol. Res..

[41] Zhang G., Chen M., Zhang S., Zhou H., Ji X., Cai J., Lou T., Cui W., Zhang N. (2018). Efficacy of caspofungin combined with trimethoprim/sulfamethoxazole as first-line therapy to treat non-HIV patients with severe pneumocystis pneumonia. Exp. Med..

[42] Tian Q., Si J., Jiang F., Xu R., Wei B., Huang B., Li Q., Jiang Z., Zhao T. (2021). Caspofungin combined with TMP/SMZ as a first-line therapy for moderate-to-severe PCP in patients with human immunodeficiency virus infection. HIV Med..

[43] Sandison T., Ong V., Lee J., Thye D. (2017). Safety and Pharmacokinetics of CD101 IV, a Novel Echinocandin, in Healthy Adults. Antimicrob. Agents Chemother..

[44] Thompson G.R., Soriano A., Skoutelis A., Vazquez J.A., Honore P.M., Horcajada J.P., Spapen H., Bassetti M., Ostrosky-Zeichner L., Das A.F. (2020). Rezafungin versus Caspofungin in a Phase 2, Randomized, Double-Blind Study for the Treatment of Candidemia and Invasive Candidiasis—The STRIVE Trial. Clinical Infectious Diseases: An Official Publication of the Infectious Diseases Society of America.

